# Supplementary figures and images for: Function and Evolution of C1-2i Subclass of C2H2-Type Zinc Finger Transcription Factors in POPLAR
Source: Genes (Basel). 2022 Oct 12;13(10):1843. doi: 10.3390/genes13101843 (PMC9602059; doi:10.3390/genes13101843)

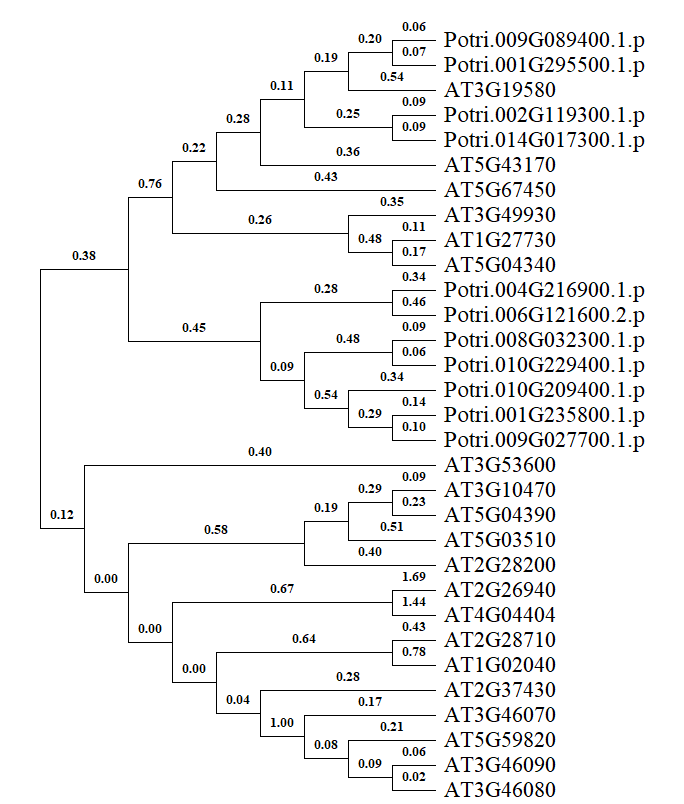

Supplement: Supplementary file 1 [file genes-13-01843-s001.zip › genes-1923170-supplementary/Supplementary files/Figure S1.tif]

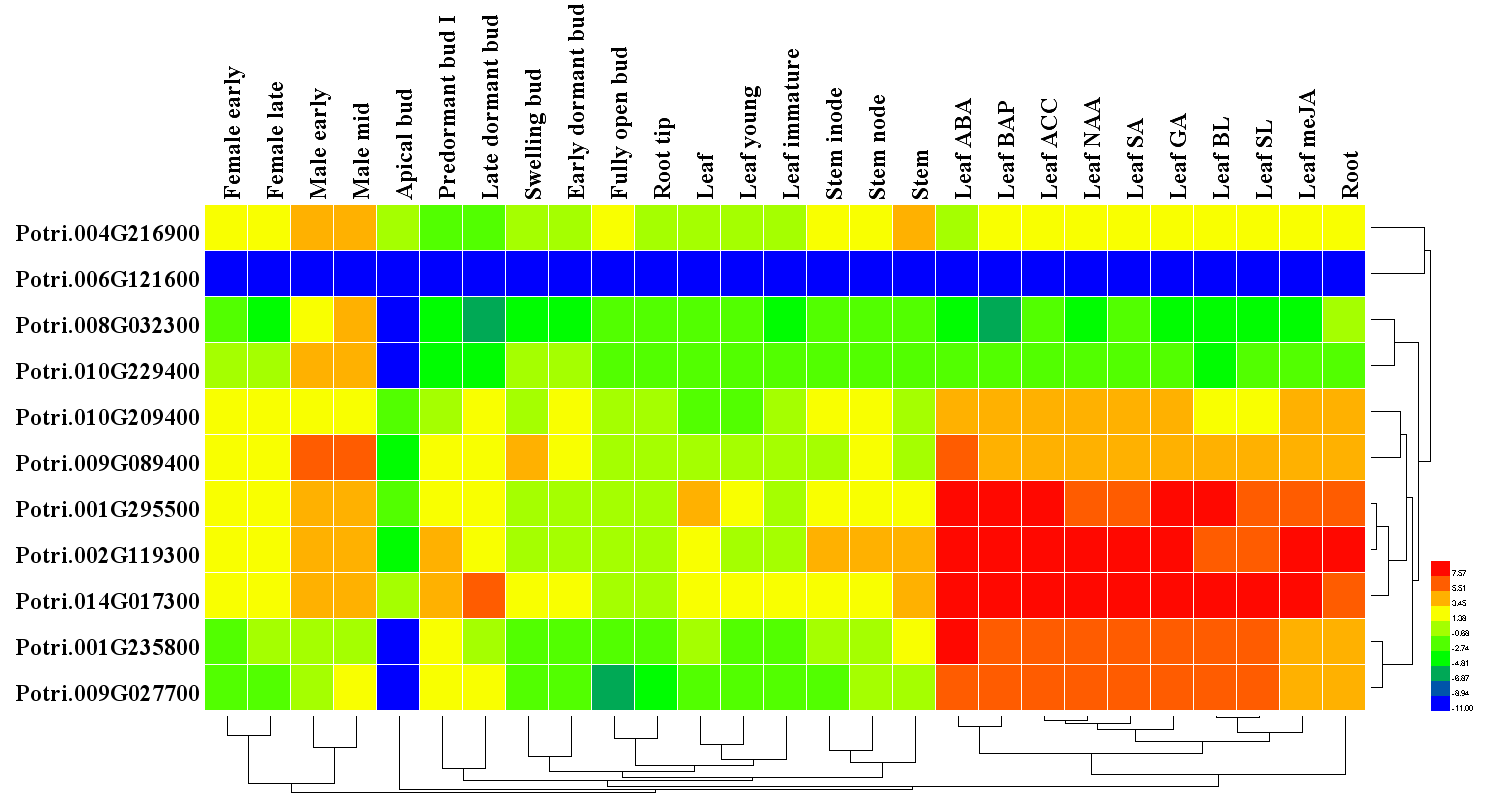

Supplement: Supplementary file 1 [file genes-13-01843-s001.zip › genes-1923170-supplementary/Supplementary files/Figure S3 .tiff]

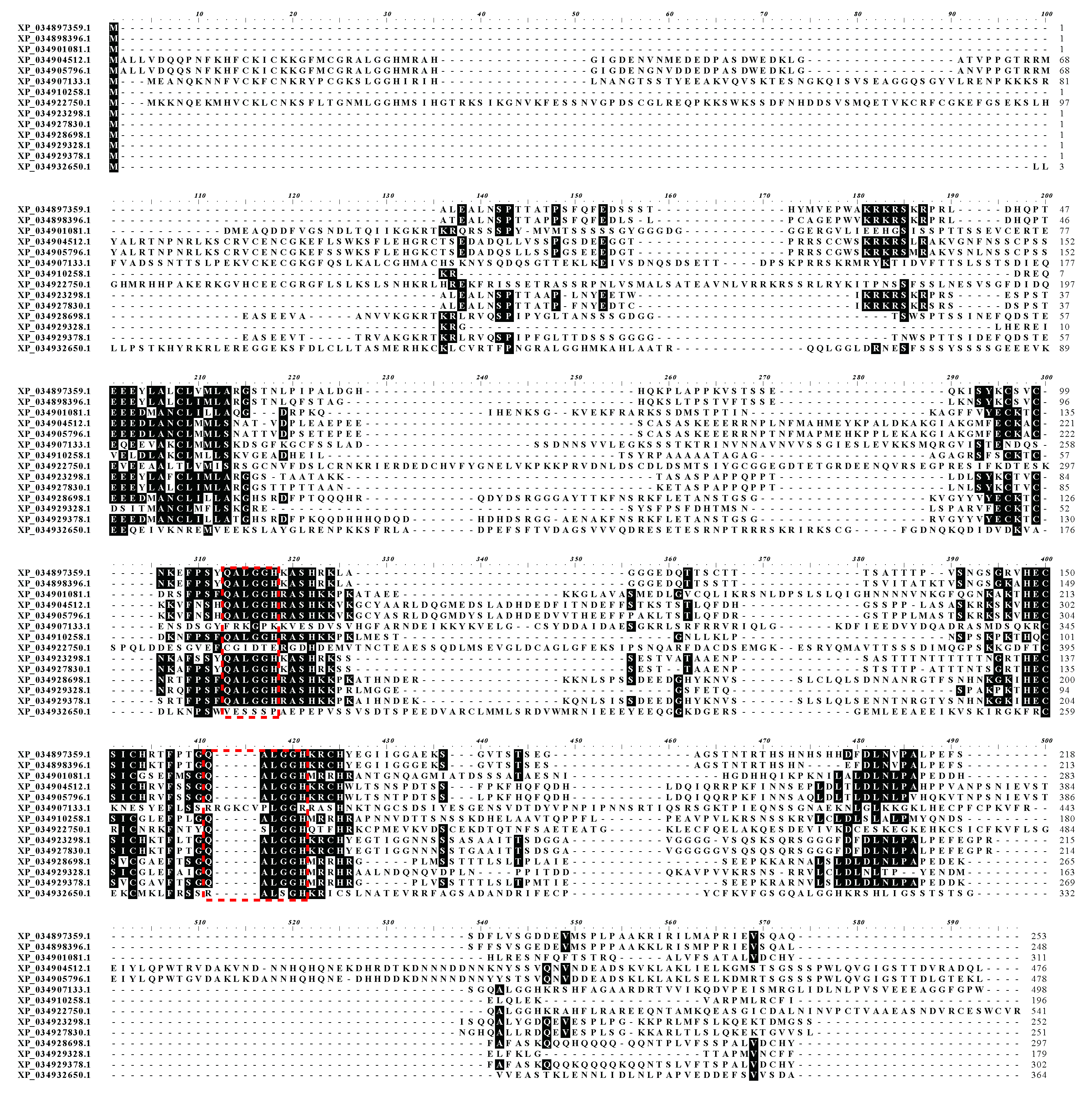

Supplement: Supplementary file 1 [file genes-13-01843-s001.zip › genes-1923170-supplementary/Supplementary files/Figure S4.tif]

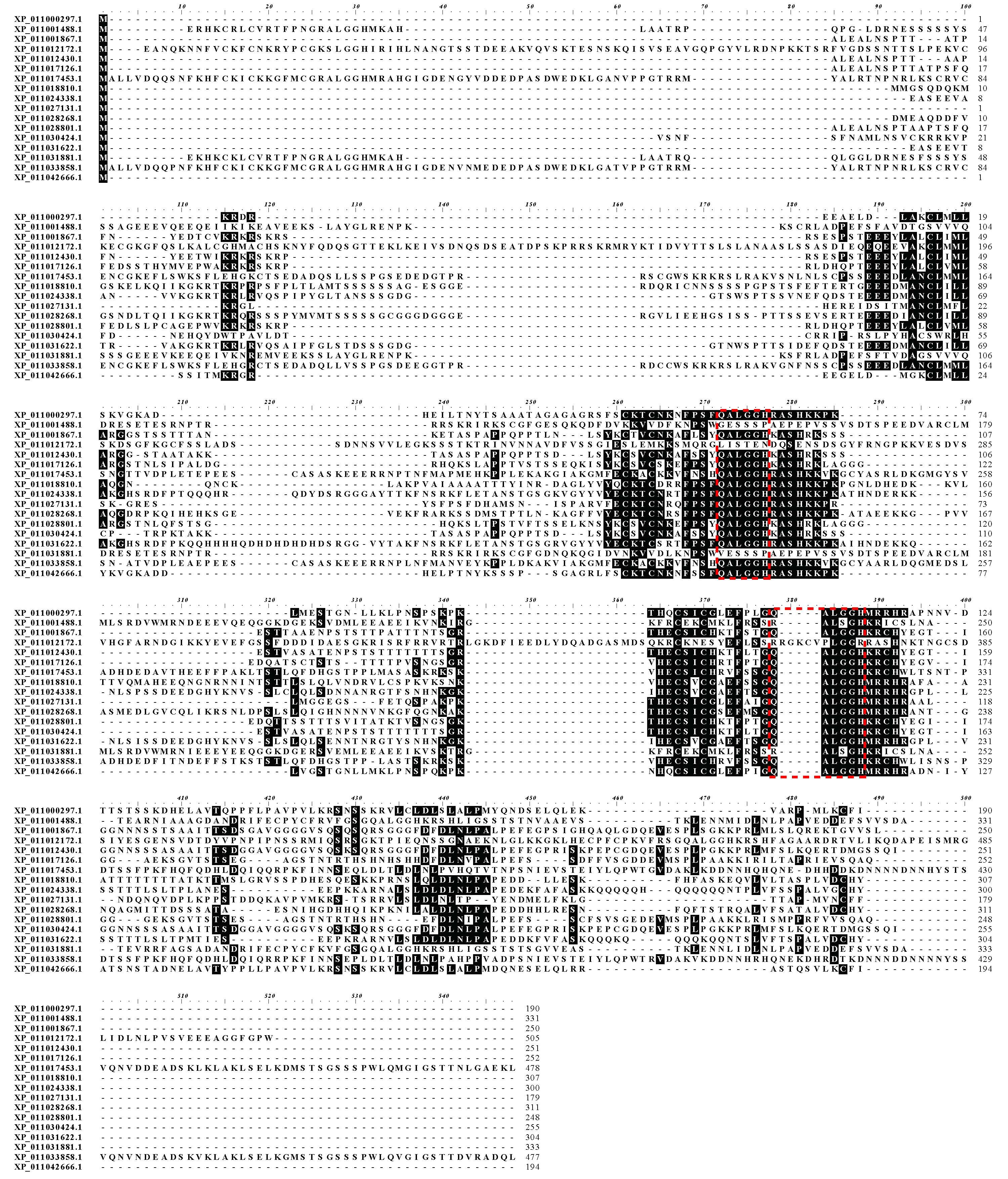

Supplement: Supplementary file 1 [file genes-13-01843-s001.zip › genes-1923170-supplementary/Supplementary files/Figure S5 .tif]

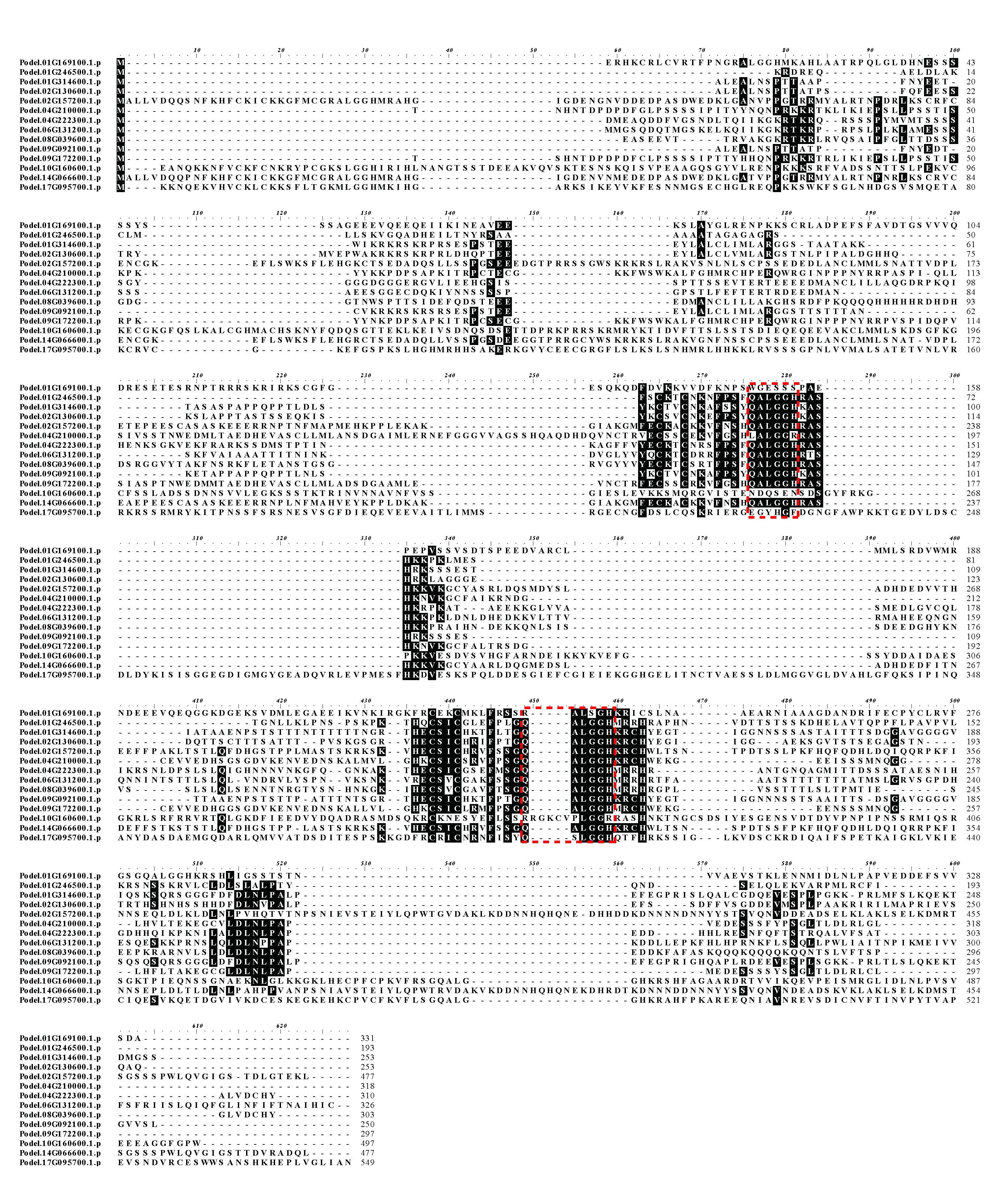

Supplement: Supplementary file 1 [file genes-13-01843-s001.zip › genes-1923170-supplementary/Supplementary files/Figure S7 .tif]
